# Methanol Assimilation in *Methylobacterium extorquens* AM1: Demonstration of All Enzymes and Their Regulation

**DOI:** 10.1371/journal.pone.0013001

**Published:** 2010-10-01

**Authors:** Hana Šmejkalová, Tobias J. Erb, Georg Fuchs

**Affiliations:** Mikrobiologie, Fakultät für Biologie, Albert-Ludwigs-Universität Freiburg, Freiburg, Germany; Cinvestav, Mexico

## Abstract

**Background:**

*Methylobacterium extorquens* AM1 is an aerobic facultative methylotrophic α-proteobacterium that can use reduced one-carbon compounds such as methanol, but also multi-carbon substrates like acetate (C_2_) or succinate (C_4_) as sole carbon and energy source. The organism has gained interest as future biotechnological production platform based on methanol as feedstock.

**Methodology/Principal Findings:**

We present a comprehensive study of all postulated enzymes for the assimilation of methanol and their regulation in response to the carbon source. Formaldehyde, which is derived from methanol oxidation, is assimilated via the serine cycle, which starts with glyoxylate and forms acetyl-CoA. Acetyl-CoA is assimilated via the proposed ethylmalonyl-CoA pathway, which thereby regenerates glyoxylate. To further the understanding of the central carbon metabolism we identified and quantified all enzymes of the pathways involved in methanol assimilation. We observed a strict differential regulation of their activity level depending on whether C_1_, C_2_ or C_4_ compounds are used. The enzymes, which are specifically required for the utilization of the individual substrates, were several-fold up-regulated and those not required were down-regulated. The enzymes of the ethylmalonyl-CoA pathway showed specific activities, which were higher than the calculated minimal values that can account for the observed growth rate. Yet, some enzymes of the serine cycle, notably its first and last enzymes serine hydroxymethyl transferase and malate thiokinase, exhibit much lower values and probably are rate limiting during methylotrophic growth. We identified the natural C_1_ carrying coenzyme as tetrahydropteroyl-tetraglutamate rather than tetrahydrofolate.

**Conclusion/Significance:**

This study provides the first complete picture of the enzymes required for methanol assimilation, the regulation of their activity levels in response to the growth substrate, and the identification of potential growth limiting steps.

## Introduction

*Methylobacterium extorquens* AM1 is an aerobic facultative methylotrophic α-proteobacterium, which can use reduced one-carbon compounds such as methanol as sole carbon and energy source. It can also utilize multi-carbon substrates like acetate, ethanol, and ethylamine (C_2_), pyruvate (C_3_), or succinate (C_4_). This metabolic flexibility seems to enable optimal adaptation to its ecological niches such as the plant phyllosphere, where *Methylobacterium* species are highly abundant with 10^4^–10^7^ colony forming units per gram of fresh plant material [1]. The release of methanol from pectin during plant growth [2] represents an important carbon source for *Methylobacterium*, but multi-carbon compounds also support plant colonization [3]–[5].

Besides its ecological relevance, *M. extorquens* has gained interest as a target for biotechnological applications, because methanol may play a major role as future alternative carbon source [6]. Since the production of single cell protein from methanol on an industrial scale in the 1960s, metabolic engineering and targeted design of industrial strains have opened new possibilities for methanol based biotechnological processes. *M. extorquens* could thus play an important role as production platform for bulk and fine chemicals. However, any potential biotechnological application of this organism requires detailed knowledge of the central carbon metabolism and its enzymatic activities.

**Figure 1:**
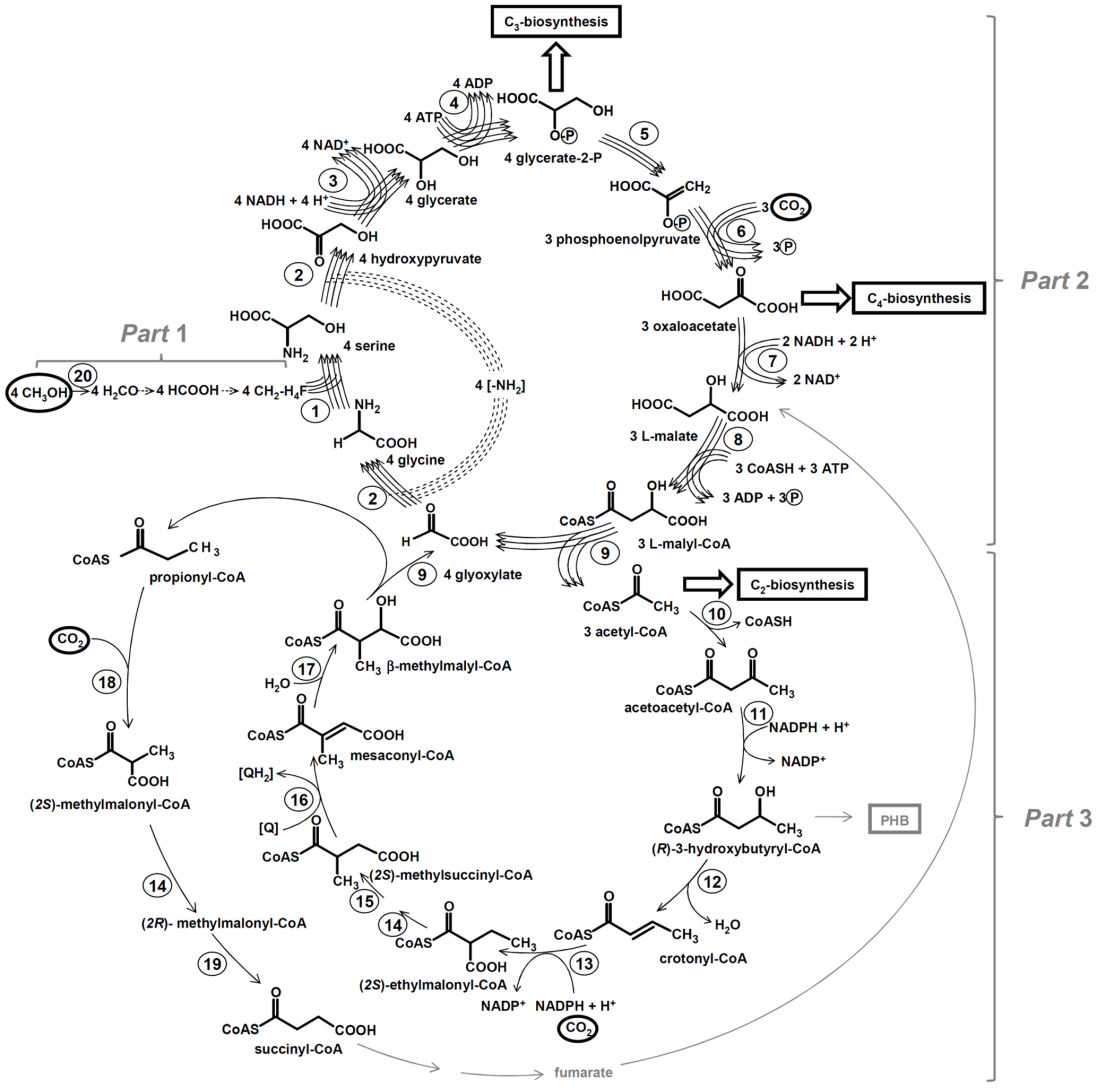
Scheme of C_1_ metabolism of the methylotroph *Methylobacterium extorquens* AM1. The bacterium oxidizes methanol to formaldehyde that is condensed with a tetrahydromethanopterin and further oxidized to formate. Formate reacts with tetrahydropterin and formyl-tetrahydrofolate is further converted to methylenetetrahydrofolate (part 1 of metabolism). The serine cycle is used for the assimilation of formaldehyde plus bicarbonate (part 2). Acetyl-CoA assimilation and conversion to glyoxylate proceeds *via* the ethylmalonyl-CoA pathway (part 3). The main biosynthetic outputs from these pathways are indicated. Enzymes: 1, serine hydroxymethyl transferase; 2, serine-glyoxylate aminotransferase; 3, hydroxypyruvate reductase; 4, glycerate kinase; 5, enolase; 6, phosphoenolpyruvate carboxylase; 7, malate dehydrogenase; 8, malate-CoA ligase (malate thiokinase); 9, L-malyl-CoA/β-methylmalyl-CoA lyase; 10, β-ketothiolase; 11, acetoacetyl-CoA reductase; 12, crotonase (*R*-specific); 13, crotonyl-CoA carboxylase reductase; 14, ethylmalonyl-CoA/methylmalonyl-CoA epimerase; 15, ethylmalonyl-CoA mutase; 16, methylsuccinyl-CoA dehydrogenase; 17, mesaconyl-CoA hydratase; 18, propionyl-CoA carboxylase; 19, methylmalonyl-CoA mutase; 20, methanol dehydrogenase. PHB, polyhydroxybutyrate, Q, quinone.

The assimilation of methanol and related C_1_ compounds in *M. extorquens* proceeds *via* three steps (Fig. 1). (1) In the periplasm methanol is oxidized to formaldehyde that enters the cell and reacts enzymatically with the C_1_ carrier coenzyme tetrahydromethanopterin [7], [8]. Formaldehyde in the form of methylene-tetrahydromethanopterin is oxidized to formate, which is either oxidized to CO_2_ yielding additional reducing equivalents or is enzymatically condensed with tetrahydrofolate to 5-formyl-tetrahydrofolate [9]–[12]. Formyl-tetrahydrofolate is further reduced *via* several enzymatic reactions to methylene-tetrahydrofolate. (2) Methylene-tetrahydrofolate is assimilated into precursors of cell material *via* the serine cycle. In this cycle, methylene-tetrahydrofolate condenses with glycine, which is formed from glyoxylate, to form serine. Serine is converted to glycerate-2-phosphate and phosphoenolpyruvate (PEP), which is carboxylated to oxaloacetate. Some of these C_3_ and C_4_ intermediates drain off the cycle for biosynthesis [12], [13]. Residual oxaloacetate is converted to malyl-CoA, which is cleaved into acetyl-CoA and glyoxylate. Owing to the deduction of biosynthetic precursors, the serine cycle can only partly regenerate the initial acceptor molecule glycine from this glyoxylate. (3) The missing part of glyoxylate is regenerated from acetyl-CoA through the so-called ethylmalonyl-CoA pathway. This pathway, which has only recently been elucidated in *Rhodobacter sphaeroides*, has been proposed to function in *Methylobacterium* [14], [15] based on earlier mutant studies [16], [17] as well as studies with recombinant enzymes [14], [15], [18], [19]. A detailed metabolome analysis recently verified its intermediates [13]. Figure 1 presents the details and an approximate mass balance of C_1_ metabolism.

Although much efforts has been made to understand the physiology of *M. extorquens* based on genomic, metabolic, proteomic, and transcriptional studies [5], [20]–[24], several enzyme activities have not been demonstrated yet. Furthermore, a comparative and complete study of all enzymes of the central carbon metabolism under different growth conditions has been lacking so far. Such a study is indispensable for identifying important factors and limiting steps in the assimilation of one-carbon as well as multi-carbon compounds.

This study aimed at demonstrating and quantifying all enzyme activities of the central carbon assimilation in *M. extorquens* grown on three representative substrates, methanol (C_1_), acetate (C_2_), and succinate (C_4_). We also identified the nature of the C_1_ carrying coenzyme. For the first time we can draw a complete metabolic picture on the enzymatic level, which allows us to unravel regulatory patterns as well as potentially rate-determining metabolic steps.

## Results and Discussion

### Enzymes of central carbon metabolism during C_1_ assimilation

**Figure 2:**
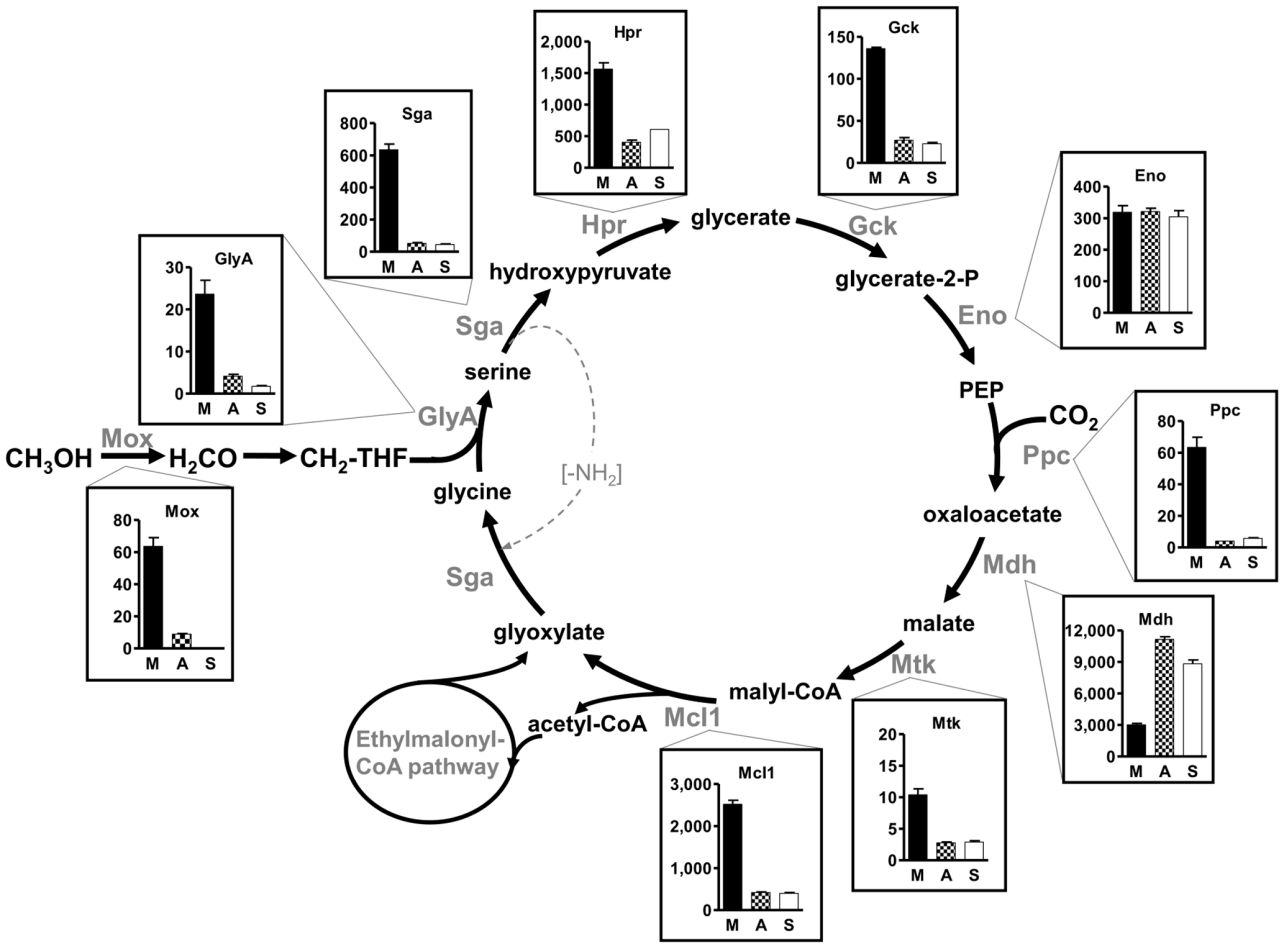
Specific activity of enzymes of the serine cycle and their regulation. Enzymes: Mox, methanol dehydrogenase; GlyA, serine hydroxymethyl transferase; Sga, serine-glyoxylate aminotransferase; Hpr, hydroxypyruvate reductase; Gck, glycerate kinase; Eno, enolase; Ppc, phosphoenolpyruvate carboxylase; Mdh, malate dehydrogenase; Mtk, malate-CoA ligase; Mcl1, malyl-CoA/β-methylmalyl-CoA lyase. The y axis is in nmol min^−1^ mg^−1^ protein. M, methanol grown cells; A, acetate grown cells; S, succinate grown cells.

Based on the proposed scheme of the central carbon metabolism (see Fig. 1), assays for all enzymatic reactions presumably involved in C_1_ assimilation in *M. extorquens* were established and used to quantify activities in extracts of cells grown with methanol as sole carbon source (generation time t_d_ = 3 h). Besides methanol dehydrogenase, all enzymatic activities of the serine cycle and the ethylmalonyl-CoA pathway could be demonstrated (Table 1), which clearly corroborates the operation of the latter pathway during C_1_-assimilation, as has been demonstrated in prior studies [13], [23]. In methylotrophic cells, methanol dehydrogenase as well as enzymatic activities of the serine cycle were in general three- to seven-fold induced compared to cells grown on multi-carbon substrates (C_2_ and C_4_) (Fig. 2, Table 1). This is consistent with known transcriptional regulation of enzymes involved in primary C_1_ assimilation (serine cycle), which is in good accordance with recent proteomic and transcriptional studies [21]–[24]. However, a post-transcriptional regulation is also possible. The only exceptions to this regulatory pattern observed were enolase and malate dehydrogenase. Both proteins are in fact housekeeping enzymes, which not only serve in methanol assimilation, but simultaneously also function in glycolysis/gluconeogenesis and citric acid cycle, respectively. Therefore, one can expect a regulation of these enzymes distinct from the general pattern. In fact, the enzyme activities obtained for these two housekeeping enzymes demonstrated that the comparison of the different cell batches was experimentally sound, despite varying growth rates of the cell cultures.

**Table 1:** Specific activities of methanol dehydrogenase, of enzymes of the serine cycle and of the ethylmalonyl-CoA pathway in extracts of *M. extorquens* cells grown on methanol, acetate or succinate.

	Specific activity [nmol min^−1^(mg protein)^−1^]*			Requirement for growth on
	Methanol	Acetate	Succinate	
	This work/previous work	This work/previous work	This work/previous work	
Methanol dehydrogenase	**64±12**/70 [25], 540 [26], 320 [27], 64 [28]	**9±0.7**/n.d.#	**<1**/60 [26], 5 [27]	C_1_
**SERINE CYCLE**				
Serine hydroxymethyl transferase	**24±7**/30 [29], 42 [30], 60 [31]	**4±0.6**/n.d.	**2±0.2**/5 [29], 13 [30]	C_1_
Serine-glyoxylate aminotransferase	**630±80**/83 [26], 120 [27], 165 [28]	**52±11**/n.d.	**45±5**/0 [26], 15 [27]	C_1_
Hydroxypyruvate reductase	**1,500±300**/1,533 [26], 343 [28], 1,200 [29], 1,570 [30], 2,250 [32]	**400±86**/n.d.	**600±10**/266 [26], 330 [29], 350 [30]	C_1_
Glycerate kinase	**130±3**/52 [26], 36 [27], [33], 11 [28], 40 [34]	**27±6**/n.d.	**23±3**/16 [26], 14 [27], [33]	C_1_
Enolase	**320±60**/n.d.	**320±18**/n.d.	**300±55**/n.d.	C_1_,C_2_,C_4_
Phospoenolpyruvate carboxylase	**63±18**/56 [27], 16 [28]	**4±<1**/n.d.	**6±1**/8 [27]	C_1_
Malate dehydrogenase	**3,000±350**/913 [30], 600 [35]	**11,000±450**/n.d.	**8,825±714**/1,263 [30], 1,616 [35]	C_1_,C_2_,C_4_
Malate-CoA ligase	**10±2**/40 [29], 50 [36]	**3±0.3**/n.d.	**3±0.3**/n.d.	C_1_
Malyl-CoA/β-methylmalyl-CoA lyase^1^	**2,500±230**/440 [27], 1,650 [37]	**410±40**/n.d.	**400±50**/80 [27], 300 [37]	C_1_,C_2_
**ETHYLMALONYL-CoA PATHWAY**				
β-Ketothiolase	**100±5**/n.d.	**390±7**/n.d.	**560±50**/392 [38]	C_1_,C_2_,C_4_
Acetoacetyl-CoA reductase	**290±50**/n.d.	**340±26**/n.d.	**390±14**/13 [38]	
Crotonase	**80±13**/n.d.	**87±5**/n.d.	**76±2**/25 [38]	
Crotonyl-CoA carboxylase/reductase	**570±80**/800 [14]	**240±0**/n.d.	**190±20**/200 [14]	C_1_,C_2_
Ethylmalonyl-CoA mutase	**73±10**/n.d.	**17±1**/n.d.	**13±2**/n.d.	C_1_,C_2_
Methylsuccinyl-CoA dehydrogenase	**160±20**/n.d.	**40±2**/n.d.	**13±2**/n.d.	C_1_,C_2_
Mesaconyl-CoA hydratase	**210±50**/n.d.	**180±0**/n.d.	**52±22**/n.d.	C_1_,C_2_
Malyl-CoA/β-methylmalyl-CoA lyase^2^	**650±60**/n.d.	**130±10**/n.d.	**86±8**/n.d.	C_1_,C_2_
Propionyl-CoA carboxylase	**74±2**/26 [17]	**49±3**/n.d.	**49±2**/n.d.	C_1_,C_2_,C_4_
Methylmalonyl-CoA mutase	**74±9**/280 [39]	**45±5**/n.d.	**32±3**/8–16 [40]	
Malyl-CoA thioesterase	**26±12**/n.d.	**120±75**/n.d.	**61±20**/n.d.	C_2_

*The values shown are means ± standard deviations of results from at least three independent measurements.

# n. d. = not determined.

1 cleavage of malyl-CoA;

2 cleavage of β-methylmalyl-CoA.

**Figure 3:**
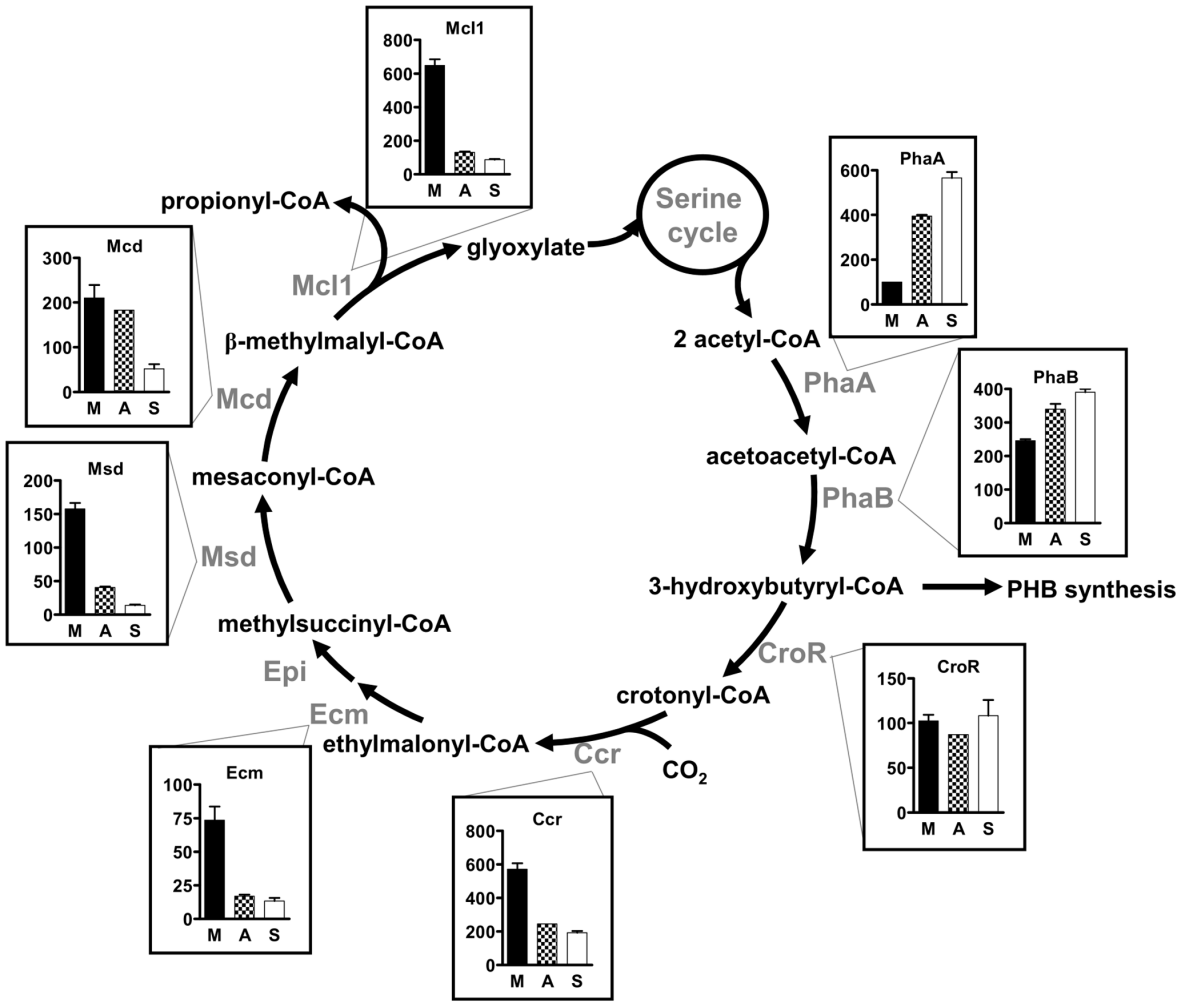
Specific activity of enzymes of the ethylmalonyl-CoA pathway and their regulation. Enzymes: PhaA, β-ketothiolase; PhaB, acetoacetyl-CoA reductase; CroR, crotonase; Ccr, crotonyl-CoA carboxylase/reductase; Epi, ethylmalonyl-CoA/methylmalonyl-CoA epimerase; Ecm, ethylmalonyl-CoA mutase; Msd, methylsuccinyl-CoA dehydrogenase; Mcd, mesaconyl-CoA hydratase; Mcl1, malyl-CoA/β-methylmalyl-CoA lyase. The y axis is in nmol min^−1^ mg^−1^ protein. M, methanol grown cells; A, acetate grown cells; S, succinate grown cells.

**Figure 4:**
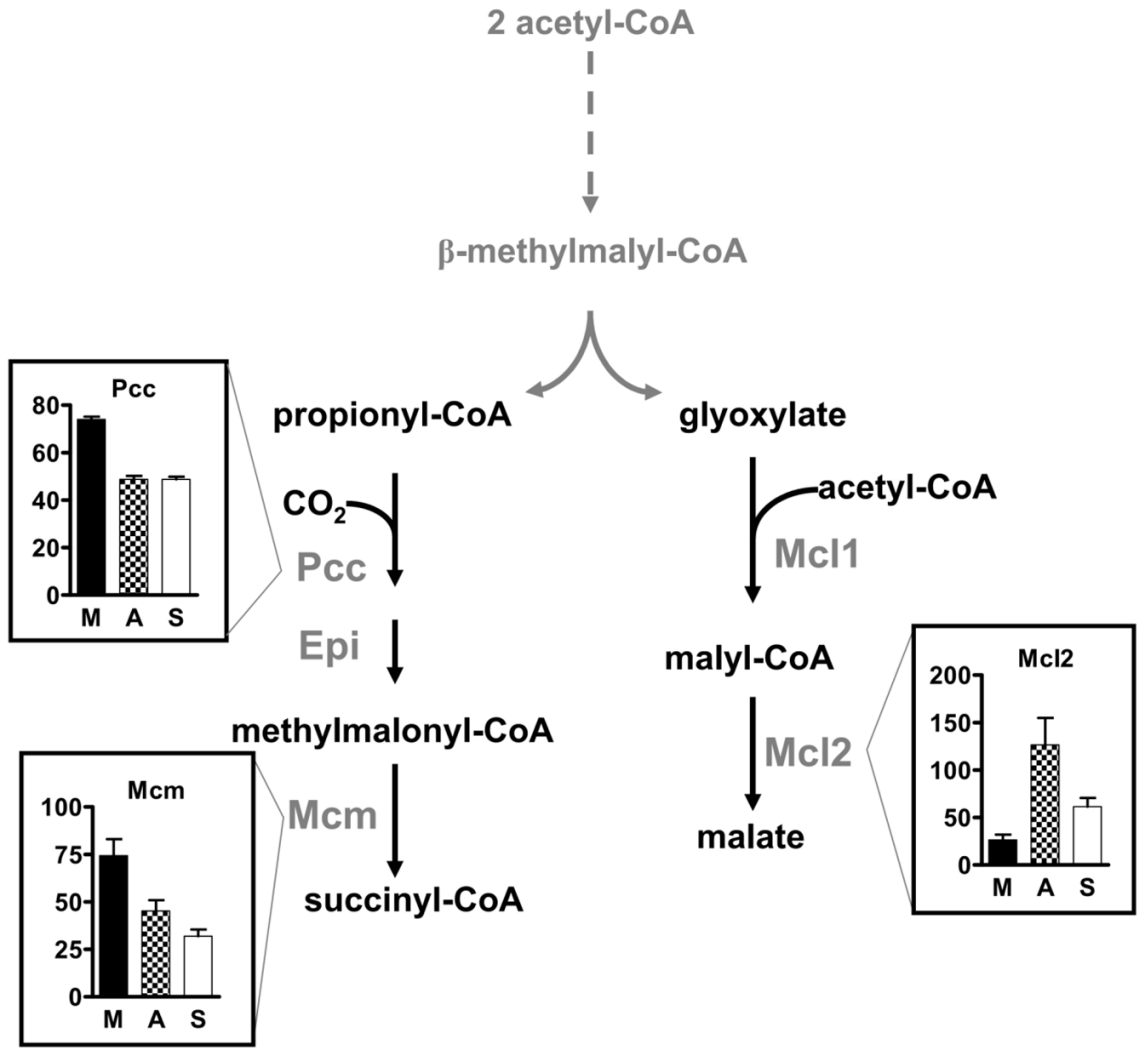
Specific activity of enzymes of the C_2_ specific steps of the ethylmalonyl-CoA pathway and their regulation. Enzymes: Pcc, propionyl-CoA carboxylase; Epi, ethylmalonyl-CoA/methylmalonyl-CoA epimerase; Mcm, methylmalonyl-CoA mutase; Mcl2, malyl-CoA thioesterase. The y axis is in nmol min^−1^ mg^−1^ protein. M, methanol grown cells; A, acetate grown cells; S, succinate grown cells.

In contrast to the serine cycle, regulation of enzymatic activities in the ethylmalonyl-CoA pathway followed a more elaborate pattern (Fig. 3). The first enzymes of the ethylmalonyl-CoA pathway leading from two molecules of acetyl-CoA to 3-hydroxybutyryl-CoA are also involved in the synthesis of the storage compound polyhydroxybutyrate (PHB) [38], [41], [42]. Their presence under all growth conditions indicates a constitutive flux of carbon through this reaction sequence. Whereas no apparent differences in activity were observed for acetoacetyl-CoA reductase and crotonase, β-ketothiolase showed higher activities in cells grown with multi-carbon substrates. In contrast to that, enzymatic steps beyond PHB synthesis, which are required for carbon assimilation and are specific for the ethylmalonyl-CoA pathway [15], were generally up-regulated in methanol grown cells by a factor five to ten (see below). Similar values have also been observed in proteomic studies [23] suggesting a high carbon flux through this reaction sequence in methylotrophic cells compared to cells grown on multi-carbon sources. Finally, the last steps of the ethylmalonyl-CoA pathway that include the carboxylation of propionyl-CoA to methylmalonyl-CoA and its subsequent conversion to succinate were constitutively expressed under all three growth conditions (Fig. 4). Since these steps are shared by fatty acid metabolism [43], a constitutive expression can be expected for this part of the pathway.

### Enzymes of central carbon metabolism during C_2_ assimilation

*M. extorquens* does not possess a functioning glyoxylate cycle and therefore the assimilation of C_2_ compounds has remained enigmatic for a long time [12]. However, with the elucidation of the ethylmalonyl-CoA pathway for acetyl-CoA assimilation in *R. sphaeroides*, this reaction sequence was proposed to operate in *M. extorquens* not only during C_1_ but also C_2_ assimilation [14], [15]. Whereas a recent metabolome study demonstrated the operation of the ethylmalonyl-CoA pathway under methylotrophic conditions [13], its operation and regulation during C_2_ assimilation was hypothetical. We therefore assayed for enzymatic activities of methanol dehydrogenase, serine cycle and ethylmalonyl-CoA pathway enzymes in extracts of cells grown on acetate as sole carbon source (t_d_ = 17 hours).

Methanol dehydrogenase, as well as enzymatic activities of the serine cycle (with the exception of housekeeping enzymes, see above) were clearly down-regulated in cells grown on acetate compared to methylotrophic cells (Fig. 2, Table 1). This was expected, since growth on acetate relies neither on C_1_ oxidation (methanol dehydrogenase) nor on C_1_ assimilation (serine cycle). In case of the ethylmalonyl-CoA pathway, pathway specific enzyme activities [15] were significantly lowered compared to methylotrophic cells, indicating a lower flux of carbon under this condition through that part of the ethylmalonyl-CoA pathway. Note that although most of the enzymes were down-regulated three- to four-fold, the residual activities might still be sufficient for growth on acetate, because of the several fold prolonged generation time (t_d_ = 17 h versus 3 h). When acetate grown cells are compared to cells grown on the C_4_ substrate succinate, some of the ethylmalonyl-CoA pathway specific enzymes were up-regulated by a factor of two to three (methylsuccinyl-CoA dehydrogenase, mesaconyl-CoA hydratase, β-methylmalyl-CoA lyase), whereas others were unaffected by the growth substrates. Similar results were obtained in a recent proteomic study that showed two-fold up-regulation of most of the ethylmalonyl-CoA pathway enzymes (except mesaconyl-CoA hydratase) during growth on ethylamine (C_2_) compared to succinate (C_4_) [44].

Another significant difference between cells grown on C_1_ and multi-carbon compounds was the strong up-regulation of malyl-CoA thioesterase [19]. During growth on acetate, combination of this enzyme with malyl-CoA lyase constitutes an apparent malate synthase activity that liberates malate from the final assimilation product malyl-CoA formed by the ethylmalonyl-CoA pathway [19], [45]. Under methylotrophic conditions malyl-CoA thioesterase is not required and would even be deleterious, since malyl-CoA is the starting point (and not the final product) of the assimilatory pathway. Hence, the strict down-regulation of this enzyme under methylotrophic conditions can be explained very well.

### Enzymes of central carbon metabolism during C_4_ assimilation

Succinate enters the central carbon metabolism on the level of the citric acid cycle, without the operation of any special assimilatory pathway [46], [47]. Intermediates can drain off this cycle directly and can be transformed into any biosynthetic precursor molecule. According to this, reactions of neither the serine cycle nor the ethylmalonyl-CoA pathway should be required by cells grown on succinate as sole carbon source.

Such a strict regulation was observed for methanol dehydrogenase as well as the serine cycle enzymes, which were in general down-regulated five- to ten-fold in succinate grown cells compared to methylotrophic cells (initial t_d_ = 3 hours; for later growth phase, the pH was adjusted by adding succinic acid which led to an increased doubling time of 10 hours). In case of the ethylmalonyl-CoA pathway, the formation of enzymes shared with PHB synthesis was basically unaffected (or even up-regulated in case of β-ketothiolase), indicating a significant carbon flux in the direction of PHB synthesis. Enzymes beyond this reaction sequence, which are specific for carbon assimilation *via* the ethylmalonyl-CoA pathway, were clearly down-regulated compared to methylotrophic cells. Compared to acetate grown cells that require the ethylmalonyl-CoA pathway for carbon assimilation, some enzymatic activities were lowered by a factor of two to three, whereas others were not significantly affected. This actually suggests that some minor flux through the ethylmalonyl-CoA pathway is possible even in succinate grown cells that do not rely on the operation of this reaction sequence.

The (low) operation of the ethylmalonyl-CoA pathway in succinate-grown cells may best be explained by some (low) flux of carbon from PHB to the central carbon metabolism, which might actually be driven by the requirement of propionyl-CoA for the synthesis of odd-numbered fatty acids. Another explanation might be a constitutively expressed ethylmalonyl-CoA pathway in *M. extorquens* that is prepared for simultaneous (co-)assimilation of C_1_, C_2_, and C_4_ substrates. A similar effect was observed in *R. sphaeroides* that seems to assimilate simultaneously acetate (*via* the ethylmalonyl-CoA pathway) and succinate, thereby essentially lacking catabolite repression [48].

### Possible limiting steps for growth on methanol

**Figure 5:**
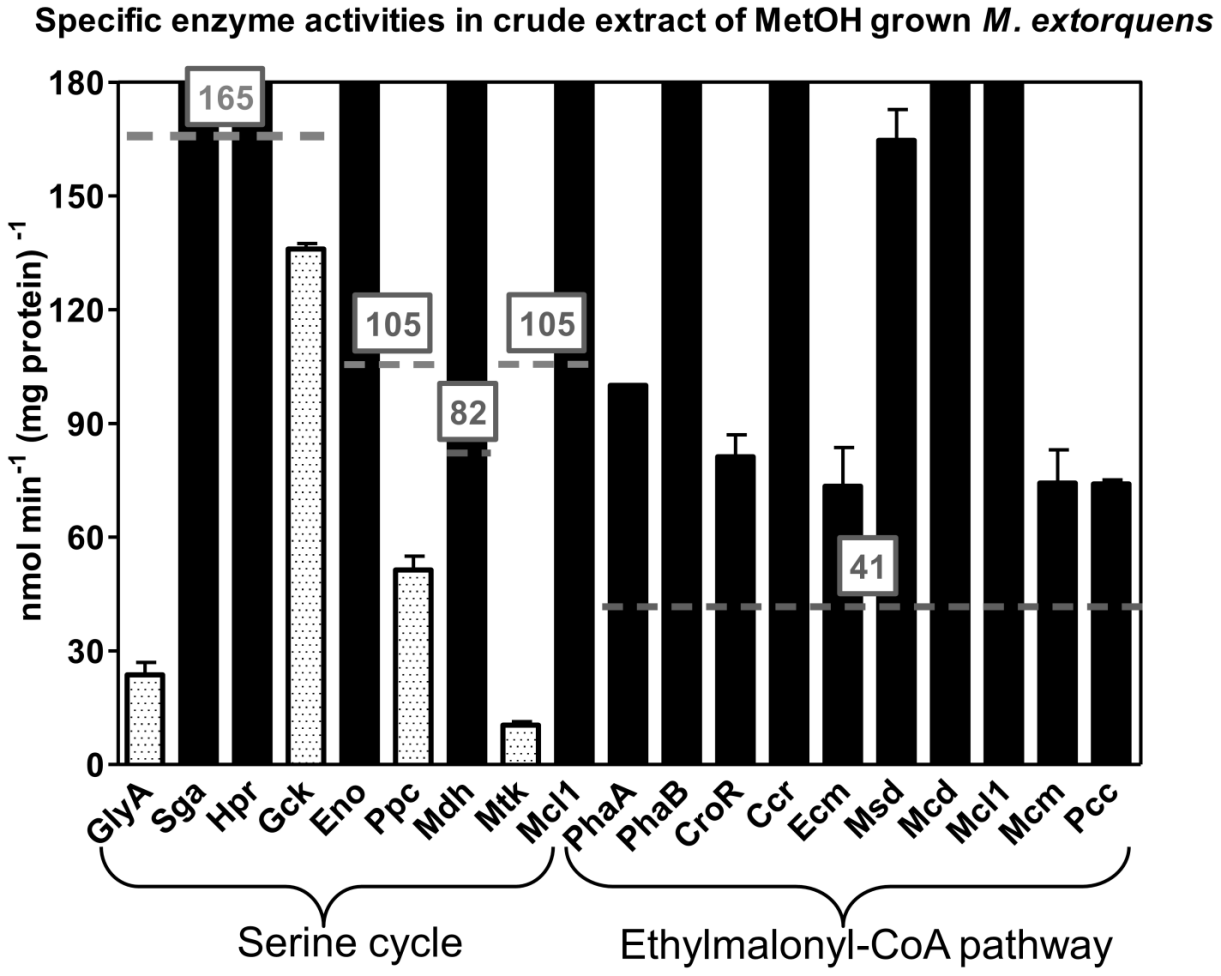
Postulated limiting steps in methanol assimilation in *M. extorquens* AM1. The grey dashed lines indicate the calculated minimal value for the specific activity of enzymes, which is required to account for the observed generation time of 3 hours on methanol.

*M. extorquens* grew methylotrophically with a generation time of 3 h, which corresponds to a specific growth rate μ of 0.231 h^−1^ requiring a specific carbon fixation rate of 330 nmol·min^−1^·mg^−1^ protein. This estimation is based on the approved equation correlating the specific substrate (S) consumption (dS) per time unit (dt) to the growth rate μ, dS/dt = (μ/Y)·X. Y represents the established growth yield for bacterial cells of 1 g of dry cell mass formed per 0.5 g of carbon fixed. Note that this figure is independent of the growth substrate and depends solely on the fact that ∼50% of bacterial cell dry mass is carbon. X refers to 1 g cell dry mass and it is a truism that in bacteria ∼50% of cell dry mass is protein; hence 1 g of cell dry mass corresponds to ∼0.5 g of protein. Assuming that two carbon atoms (formaldehyde and HCO_3_^−^) are fixed in one turn of the serine cycle, the minimal specific activity of its enzymes required *in vivo* is 165 nmol·min^−1^·mg^−1^ protein. Hypothetical minimal enzyme activities (dashed lines in Fig. 5) were calculated using this value (165 nmol·min^−1^·mg^−1^ protein) and the relative flux distributions from a ^13^C metabolomic study [13]. Activities far above that minimal value indicate that these steps are probably not rate limiting. An activity significantly below this value may indicate a potentially rate limiting step. Those enzymes are candidates for further evaluation.

According to our measurements, serine hydroxymethyl transferase, glycerate kinase (forming glycerate 2-phosphate), phosphoenolpyruvate carboxylase, and *L*-malate-CoA ligase (see below) exhibited specific activities lower than 165 nmol·min^−1^·mg^−1^ protein under methylotrophic conditions. Determination of metabolite pools [49] showed high (>10 mM) pool sizes of glyoxylate and glycine, which actually points to a limiting serine hydroxymethyl transferase. Somewhat lower pool sizes have been determined for glycerate (3 mM) and malate (1.6 mM), whereas the other metabolites of the serine cycle were in the order of 1±0.5 mM, which would be in line with a limiting role of glycerate kinase and *L*-malate-CoA ligase. The pool sizes of the CoA-linked intermediates of the ethylmalonyl-CoA are not available, but their changes in response to the growth substrate have been determined [13]. In contrast to various enzyme activities of the serine cycle, none of the enzyme activities of the ethylmalonyl-CoA pathway was below the calculated minimal enzyme activity.

### Methanol dehydrogenase

In *M. extorquens* methanol dehydrogenase is a periplasmic quinoprotein that catalyzes the oxidation of methanol to formaldehyde, the first step in this metabolism. Formaldehyde is partly oxidized to CO_2_ and partly assimilated into cell material [10]. For determination of enzyme activity we used a modified spectrophotometric assay according to [25] and found similar values in methanol grown cells (∼64 nmol min^−1^ mg^−1^) as reported before. This value cannot explain the growth rate of the bacterium, as calculated above. However, much higher specific activities (five- to ten-fold) have been reported using a different enzyme assay [26], [27] and it has been shown that the natural electron acceptor of this enzyme is in fact an unusually large, acidic cytochrome cL [50]. Use of the natural electron acceptor *in vitro* might result in higher specific activities. One would expect a tight coordination of growth rate and methanol oxidation, otherwise accumulated toxic formaldehyde and excess reducing agents would be deleterious.

### Serine hydroxymethyl transferase and re-examination of its native tetrahydrofolate cofactor

We measured serine hydroxymethyl transferase (GlyA) in the physiological direction (formation of serine from glycine and activated formaldehyde) following formation of serine with tetrahydrofolate as cofactor. Using this standard assay, the maximal measured specific activity was about 30 mU per mg protein in cell extract. Similar values were previously published in the literature: 30 mU per mg, when measured in the reverse direction [29], 42 mU per mg, when using an assay that followed the disappearance of formaldehyde [30]. All these values are dramatically lower than the theoretical limit of 165 mU per mg (Fig. 5) warning that the used assays were not optimal.

It is well known that folic cofactors (tetrahydropteroyl cofactors) exist *in vivo* as mono-glutamated (tetrahydrofolate) or as poly-glutamated species (tetrahydropterins) [51]. Enzymes transforming such C_1_ carriers may be indifferent to or highly specific for the kind of cofactor. Tetrahydropteroyl**tri**glutamate stimulated serine synthesis from glycine and formate in *M. extorquens* extracts more than tetrahydropteroyl**mono**glutamate; whereas this **tri**glutamate had no significant effect when formaldehyde was tested [30]. In order to resolve this puzzle and to identify the natural coenzyme we undertook an analysis of the native tetrahydrofolic acid cofactor.

Protein free cell extract of methanol grown *M. extorquens* was incubated with [^14^C]formaldehyde, which reacts spontaneously with uncharged C_1_ carrier molecules (tetrahydromethanopterin, tetrahydrofolic acid and poly-glutamated tetrahydropterins) to obtain the corresponding labeled methylene derivatives. Subsequent HPLC analysis of the extract showed two major radioactive peaks with distinct UV spectra (data not shown). The first eluting at 16.3 min was assigned to methylene- and methenyl-derivatives of tetrahydromethanopterin based on its specific absorption spectrum [52]. In contrast, the UV spectrum of the second radioactive compound eluting at 22.3 min resembled standard methylene-tetrahydrofolate (tetrahydropteroyl**mono**glutamate) with a maximum at 286 nm, but the retention time differed (standard methylene-tetrahydrofolate, 20.8 min). This unknown radioactive substance was therefore supposed to be a poly-glutamated methylene-tetrahydropterin [53].

To isolate the native cofactor, methanol grown cells were fed with trace amounts of [*carboxy*-^14^C]4-aminobenzoic acid (0.17 µM), the direct precursor of pterin cofactors. Substantial incorporation of radioactivity in the cells (25%) was determined by liquid scintillation counting. Pterin fractions (radioactive fractions with an absorption maximum at 286 nm) were isolated from protein free cell extracts using semi-preparative HPLC and subjected to mass spectrometric analysis. Electron spray ionization mass spectroscopy (ESI-MS) showed the presence of 5-formyl-tetrahydropteroyl **tetra**glutamate (M = 860.3) ions in the negative mode m/z = 859.3 (76%, [M-H]^+^) and also in the positive mode m/z = 861.2 (7%, [M+H]^+^). However, due to the high sensitivity of pterins to light and oxygen [54] several decomposition products were also detected. These included *p*-aminobenzoyl-glu_4_ acid (m/z = 653.3); *N*-methyl-*p*-aminobenzoyl-glu_4_ acid (m/z = 667.3); *N*-formyl-*p*-aminobenzoyl-glu_4_ acid (m/z = 681.3); and *N*-hydroxymethyl-*N*-methyl-*p*-aminobenzoyl-glu_4_ acid (m/z = 697.3), all in the ESI negative mode.

In conclusion, these experiments indicate that tetrahydropteroyl**tetra**glutamate acts as the native cofactor of serine hydroxymethyl transferase, which may explain the low activities *in vitro* that had been determined using tetrahydrofolate (tetrahydropteroyl**mono**glutamate) as cofactor. This assumption was corroborated by enzyme assays. Application of the isolated native cofactor instead of tetrahydrofolate led to a two-fold increase of the serine hydroxymethyl transferase activity. This effect may even be bigger since the decomposition products of the labile cofactor might have inhibited the assay [55].

### Glycerate kinase, phosphoenolpyruvate carboxylase, and malate thiokinase

Very low activities of glycerate kinase (Gck) have been reported before (11–36 mU per mg protein) [27], [28], [33]. However, the specific activity of glycerate kinase measured in this study was about 130 mU per mg protein, which is close to the calculated minimal requirement (165 mU per mg) (Table 1, Fig. 5). Also the activity of phosphoenolpyruvate carboxylase (Ppc) of about 60 mU per mg protein (56 mU per mg reported before [27]) represented only half of the theoretical minimal value (Table 1, Fig. 5).

The activity of malate thiokinase (Mtk) (*L*-malate-CoA ligase (ADP forming)) was measured using a continuous assay with recombinant *L*-malyl-CoA lyase Mcl1 cleaving the reaction product (malyl-CoA) into acetyl-CoA and glyoxylate (glyoxylate being determined spectrophotometrically at 324 nm as phenylhydrazone). The specific activity in methanol grown cells was only about 10 mU per mg protein (Table 1, Fig. 5). All attempts to measure malate activation with different nucleoside triphosphates (GTP, UTP) or CoA donors (succinyl-CoA) were unsuccessful (data not shown). Higher activities (50 mU per mg protein) have been reported using a coupled assay for ADP detection (with pyruvate kinase and lactate dehydrogenase as auxiliary enzymes) [36], and 40 mU per mg protein using an assay coupled to *L*-malyl-CoA lyase and spectrophotometric acetyl-CoA detection at 232 nm [29]. In all cases, the considerably lower activity compared to the postulated value (165 mU per mg) indicates that this energetically demanding reaction is not yet fully understood and could represent a metabolic bottleneck. A metabolome study corroborates this conclusion [57]. Among monitored metabolites of the serine cycle, malate (as substrate for the thiokinase) reached the highest concentration in cells. Moreover, malate thiokinase may also be a target for enzyme regulation via acetylation/deacetylation; this phenomenon is known from AMP forming acyl-CoA synthetases [58].

### Conclusions

Our new data contribute to a more complete picture of the central carbon metabolism of *M. extorquens* by demonstrating all reactions of the ethylmalonyl-CoA pathway in methanol grown cells and by showing the regulation of all enzymes involved in response to C_1_, C_2_ and C_4_ substrates (Table 1). In addition, we identified the variant of tetrahydrofolate present in this bacterium. Former studies on the serine pathway discussed the regulation of serine cycle enzymes in *M. extorquens* AM1 only partially, and compared mainly growth on C_1_ compounds to growth on C_4_ compounds [27], [29]. Growth on C_2_ compounds has been rarely discussed [41], [56] and enzymes of the ethylmalonyl-CoA pathway were not considered. We observed significantly higher activities of the ethylmalonyl-CoA pathway enzymes in acetate grown cells compared to succinate grown cells, corroborating the role of the ethylmalonyl-CoA pathway in acetate assimilation. This finding is consistent with previous studies on mutants unable to utilize C_1_ and C_2_ substrates [17], [26], [38], [59] and with a very recent study [44].

The ethylmalonyl-CoA pathway, in connection with the serine cycle, represents an elegant solution of methanol assimilation, where methanol and carbon dioxide contribute nearly equally to cell carbon (Fig. 1): 4 C_1_ (formaldehyde)+5 CO_2_+8 NAD(P)H+8 H^+^+8 ATP+1 Coenzyme A→1 C_3_ (glycerate-2-phosphate)+1 C_4_ (oxaloacetate)+1 C_2_ (acetyl-CoA)+8 NAD(P)^+^+2 [H]+8 ADP+7 P_i_. The assimilation of acetate through the ethylmalonyl-CoA pathway can be expressed by the following equation: 3 acetyl-CoA+2 CO_2_+2 NADPH+2 H^+^+ATP→malate+succinyl-CoA+2 CoA+2 [H]+2 NADP^+^+ADP+P_i_.

We identified potential metabolic bottlenecks for the growth of *M. extorquens* on methanol, and the results of the specific activity measurements in cell extract of methanol grown cells compare well with results from the ^13^C metabolome study [13]. These critical steps are the initial C_1_-fixation reaction of the serine cycle catalyzed by serine hydroxymethyl transferase and three reactions, in which high-energy phosphate bonds are hydrolyzed (glycerate kinase, phosphoenolpyruvate carboxylase, *L*-malate-CoA ligase). These finding might point to an energy-limited lifestyle of *M. extorquens*, in which assimilation strongly correlates to the availability of ATP. Surprisingly, all potential limiting steps are reactions of the serine cycle and not of the ethylmalonyl-CoA pathway. Only under some artificial conditions (cobalt limitation [57], [60]) coenzyme-B_12_ dependent reactions of the ethylmalonyl-CoA pathway may become rate limiting.

Single enzymes involved in C_1_-fixation can actually be a growth rate limiting factor, as shown for sedoheptulose-1,7-bisphosphatase [61], an enzyme operating in the reductive pentose phosphate (Calvin) cycle, or by the low catalysis rate of ribulose 1,5-bisphosphate carboxylase/oxygenase [62]. However, this simplified concept of limiting enzymatic steps has its limitations and even flaws. On one hand, for purely technical reasons the *in vitro* activities may be biased relative to those actually occurring *in vivo*. This may be true for enzymes with in vitro activities significantly below the theoretical minimum; in this case an important stimulating factor may have been overlooked in the assay. On the other hand, enzymes with a *v_max_* value far higher than the calculated minimal activity and with a reasonably low *K_M_* value are unlikely candidates for bottlenecks. Our analysis did not take into account allosteric regulation nor *K_M_* values. There may not be a single enzymatic activity (with the lowest *v_max_*) that sets the pace for the whole cell, because enzymes at steady state hardly are saturated with substrate. Still, those enzymes with particularly low *v_max_*, as identified in this work, may very well be excellent candidates for those that exert the greatest degree of control over flux, but then the situation becomes continuous and non-linear, and cannot be captured by simply identifying a single enzyme with the lowest (*in vitro*) *v_max_*_._ Thus, other considerations could be taken into account. An alternative paradigm is that one considers the quantitative contribution of every single enzyme to the overall steady-state rate of catalysis, i.e. metabolic control analysis [63]–[69]. Yet, the results of this study will contribute to a better understanding of the physiology of *M. extorquens* and are indispensable for the ongoing efforts to model central carbon metabolism of *Methylobacterium* also in view of its biotechnological application (R. Peyraud, P. Kiefer, and J. A. Vorholt, personal communication).

## Materials and Methods

### Materials

Chemicals were obtained from Sigma-Aldrich (Taufkirchen, Germany), Merck (Darmstadt, Germany), Applichem (Darmstadt, Germany), Roth (Karlsruhe, Germany), Serva (Heidelberg, Germany) or Gerbu (Craiberg, Germany). Radiochemicals were purchased from Hartmann Analytic (Braunschweig, Germany).

### Syntheses of CoA-esters

Acetyl-CoA, crotonyl-CoA and propionyl-CoA were synthesized from the corresponding anhydrides and acetoacetyl-CoA was synthesized from diketen [70]. The (*R*)- and (*S*)-stereoisomers of 2-methylsuccinyl-CoA and of 3-hydroxybutyryl-CoA were synthesized by the mixed anhydride method [71]. *L*-malyl-CoA and β-methylmalyl-CoA were synthesized enzymaticaly using recombinant malyl-CoA lyase (Mcl1), and mesaconyl-CoA from β-methylmalyl-CoA using recombinant mesaconyl-C1-CoA hydratase from *R. sphaeroides* [19], [72].

### Bacterial strain and growth conditions

*Methylobacterium extorquens* AM1 was obtained from DSMZ (Braunschweig, Germany). *M.* ex*torquens* was grown on minimal medium according to [13] in a 10 L and 200 L fermenter, respectively. The minimal medium was supplemented with 0.5% (v/v) methanol, 10 mM acetate or 10 mM succinate as the sole sources of carbon and energy. The temperature was kept at 30°C, the pH was kept at 6.5 to 7.5 by addition of acetic acid (10 mM) and succinic acid (37 mM), respectively. In the case of methanol no pH adjustment was needed. Cells were harvested by centrifugation in the exponential growth phase (at OD_578nm_ = 2, in case of acetate at OD_578nm_ = 0.75) and stored at −70°C until use.

### Preparation of cell extracts

One part of cells (wet mass) was suspended in one volume of buffer (the same as used in enzyme assay) at usual concentrations from 10 to 50 mM containing 0.2 mg DNase I per mL of cell suspension. The cell suspension was passed twice through a chilled French pressure cell at 137 kPa. The lysate was ultracentrifuged for 1 h at 100,000×g at 4°C and the supernatant was used as cell extract. Alternatively, on a small scale cells were disrupted using a mixer mill (type MM2, Retsch, Haare, Germany). To one mL of the cell suspension (1∶1) 500 mg glass beads (diameter 0.25–0.5 mm) were added and cooled cells were disrupted by 10 min treatment at 30 Hz. The supernatant after centrifugation at 16,000×g for 30 min at 4°C was used as cell extract. Protein was determined by the Bradford method [73] using bovine serum albumin as a standard.

### Enzyme assays

Enzyme activities were assayed at 30°C using freshly prepared cell extracts. Spectrophotometric enzyme assays were done in 0.5 mL glass cuvettes, unless otherwise stated. Reactions involving NAD(P)H were measured at 365 nm (ε_NADH_ = 3.4×10^3^ M^−1^cm^−1^, ε_NADPH_ = 3.5×10^3^ M^−1^cm^−1^) [74]. Reactions with phenylhydrazine were measured at 324 nm (ε_glyoxylatephenylhydrazone_ = 17×10^3^ M^−1^cm^−1^) [74]. One unit corresponds to 1 µmol substrate converted per minute.

Methanol dehydrogenase was measured using a modified assay according to [25]. The reaction mixture (200 µL) contained 85 mM Tris/HCl buffer pH 9, 15 mM NH_4_Cl, 1 mM KCN, 0.11 mM phenazine methosulfate (PMS) and 0.8 mM 2,6-dichlorphenol-indophenol (DCPIP). The reaction was followed spectrophotometrically at 600 nm in a cuvette with a light path of 0.1 cm (ε_DCPIP_ = 22×10^3^ M^−1^cm^−1^) [74]. After addition of the cell extract the absorbance first decreased and subsequently increased again. After the endogenous reaction of the dye was over (no more change of absorbance, approximately 40 min), 5.3 mM methanol was added to start the reaction.

Enzymes of the serine cycle generally were assayed as already reported elsewhere, with only minor alterations [75]–[77].

Serine-hydroxymethyl transferase (GlyA) was assayed discontinuously using radioactive labeled glycine. The assay mixture contained 100 mM potassium/sodium phosphate pH 7.5, 0.03 mM pyridoxal phosphate, 2 mM [2 - ^14^C] glycine (185 kBq/mL of reaction mixture) and 1.2 mM methylene-tetrahydrofolic acid (synthesized as described previously [78]). The reaction was started by addition of cell extract. The reaction was stopped by direct application of the sample (4 µL) on a silica-gel thin layer chromatography plate and immediate drying. The substrate and product formed were separated using a tert-butanol/acetone/water/25% aqueous ammonia = 45∶30∶15∶10 (v/v) solvent system. Radioactivity was detected by phosphoimaging and spots for glycine and serine were isolated and quantified by liquid scintillation counting.

Enolase activity was assayed in a continuous photometric assay coupled with pyruvate kinase and lactate dehydrogenase (rabbit muscle). The reaction mixture (300 µL) contained 100 mM Tris/Cl pH 7.5, 25 mM MgSO_4_, 100 mM KCl, 0.3 mM NADH, 1.3 mM ADP, 0.7 U of pyruvate kinase, 1.2 U of lactate dehydrogenase, and cell extract. The reaction was started by addition of 2 mM 2-phosphoglycerate. The oxidation of NADH was followed.

Malate-CoA ligase (malate thiokinase, Mtk) was determined using a continuous photometric assay coupled with an excess of recombinant *L*-malyl-CoA lyase (Mcl1) from *R. sphaeroides* [19]. The reaction mixture (300 µL) contained 100 mM (*N*-morpholino)propanesulfonic acid (MOPS)/KOH pH 7.5, 10 mM MgCl_2_, 3 mM phenylhydrazine, 5 mM ATP, 20 mM *L*-malate, 1 U Mcl1, and cell extract; the reaction was started by adding 0.5 mM coenzyme A. The formation of glyoxylate phenylhydrazone was followed spectrophotometrically.

β-Ketothiolase (PhaA) was measured using a continuous photometric assay coupled with an excess of recombinant acetoacetyl-CoA reductase (PhaB) from *R. sphaeroides* [48]. The reaction mixture (300 µL) contained 100 mM Tris/Cl pH 7.8, 0.5 mM NADPH, 0.4 U of PhaB, and cell extract; the reaction was started by addition of 0.7 mM acetyl-CoA.

Acetoacetyl-CoA reductase (PhaB) was measured using a continuous photometric assay. The reaction mixture (300 µL) contained 100 mM Tris/Cl pH 7.8, 0.5 mM NADPH, and cell extract; the reaction was started by addition of 0.5 mM acetoacetyl-CoA.

Crotonase (*S*-specific enoyl-CoA hydratase, CroR) was tested in a photometric continuous assay coupled with an excess of recombinant crotonyl-CoA carboxylase/reductase (Ccr) from *R. sphaeroides*. The reaction mixture (200 µL) contained 100 mM Tris/Cl pH 7.8, 40 mM NaHCO_3_, 4 mM NADPH, 4 U of Ccr, and cell extract; the reaction was started by addition of 1 mM (*S*)-3-hydroxybutyryl-CoA. The reaction was followed in a cuvette with a path length of 0.1 cm.

Crotonyl-CoA carboxylase/reductase was assayed using a continuous photometric assay as already reported in [14].

Ethylmalonyl-CoA mutase was determined using a discontinuous radiolabeled assay. For product analysis high performance liquid chromatography (HPLC) was used as reported in [18].

Methylsuccinyl-CoA dehydrogenase was assayed by a continuous photometric assay using ferrocenium hexafluorophosphate as an artificial electron acceptor; alternatively, a discontinuous assay with HPLC analysis was used as reported in [15].

Mesaconyl-C1-CoA hydratase was determined using a continuous photometric assay coupled with an excess of recombinant Mcl1 from *R. sphaeroides* [19]. The reaction mixture (300 µL) contained 100 mM MOPS/KOH pH 7.5, 4 mM MgCl_2_, 3.5 mM phenylhydrazine hydrochloride, 1 U Mcl1, and cell extract; the reaction was started by addition of 0.2 mM mesaconyl-C1-CoA.

The cleavage of β-methylmalyl-CoA catalyzed by the bifunctional malyl-CoA/β-methylmalyl-CoA lyase (Mcl1) was assayed using a modified continuous photometric assay as used for *L*-malyl-CoA cleavage. The reaction mixture (300 µL) contained 100 mM MOPS/KOH pH 7.5, 4 mM MgCl_2_, 3.5 mM phenylhydrazine hydrochloride, and cell extract; the reaction was started by addition of 0.44 mM β-methylmalonyl-CoA instead of malyl-CoA.

Propionyl-CoA carboxylase was assayed using a radiolabelled discontinuous enzyme test. The reaction mixture (500 µL) contained 200 mM MOPS/KOH pH 8, 5 mM MgCl_2_, 5 mM dithiothreitol (DTT), 2 mM ATP, 0.4 mM propionyl-CoA, and 10 mM [^14^C] NaHCO_3_ (65 kBq); the reaction was started by addition of cell extract. Samples (100 µL) were added to a 2-fold volume of 2% trichloroacetic acid and then incubated at room temperature while shaking for 12 hours to remove volatile ^14^CO_2_. In control experiments, ATP was omitted. The remaining radioactivity in the samples was measured by liquid scintillation counting.

Methylmalonyl-CoA mutase was determined using a discontinuous assay. The reaction mixture (250 µL) contained 100 mM Tris/HCl pH 7.8, 0.5 mM coenzyme B_12_, 1 mM (*R*,*S*)-methylmalonyl-CoA (Fluka), 1 U of recombinant epimerase from *R. sphaeroides*. The reaction was started by addition of the cell extract. Samples (50 µL) were stopped by adding 5 µL of 20% formic acid and diluted with 50 µL water. The precipitated protein was removed by centrifugation. Samples were analyzed using the same HPLC method as for analysis of products of ethylmalonyl-CoA mutase [18].

*L*-Malyl-CoA thioesterase (Mcl2) [19] was measured using a discontinuous photometric test with 5,5′-dithiobis-(2-nitrobenzoic acid) (DTNB). The reaction mixture (250 µL) contained 100 mM Tris/HCl pH 7.5, 5 mM MgCl_2_, and cell extract; the reaction was started by addition of 0.1 mM *L*-malyl-CoA (free of coenzyme A). Samples (40 µL) were stopped by adding 110 µL of 3.33 mM DTNB solution (in 0.5 M MOPS/KOH pH 7.5, freshly prepared) and the linear increase of absorption at 412 nm was immediately measured (ε_(DTNB+CoA)_ = 13.6×10^3^ M^−1^cm^−1^) [74].

### Labeling of C1 carrier molecules in protein free cell extract of *M. extorquens* with [^14^C]formaldehyde

One part of frozen cells (wet mass) of *M. extorquens* grown on methanol was suspended under anaerobic conditions in one volume ice cold extraction buffer containing 5% (v/v) 75 mM KH_2_PO_4_, 52 mM sodium ascorbate, 10 mM 2-mercaptoethanol buffer pH 6 and 95% (v/v) methanol. The suspension was passed twice through an anaerobic chilled French pressure cell at 137 kPa and centrifuged for 20 min at 16,000×g and 4°C to remove precipitated protein. The supernatant was concentrated by vacuum evaporation and freeze-drying. The freeze-dried sample was dissolved in a small amount of water and centrifuged. To label one carbon carrier molecules the highly concentrated protein-free cell extract (50 µL) was incubated with 18.5 kBq of [^14^C]formaldehyde (9 nmol) at room temperature under N_2_ atmosphere. After 20 min of incubation the pH of the solution was adjusted to 9 with 1 M NaOH. The labeled low molecular mass compounds were analyzed by HPLC with UV detection (Waters 996, PDA Detector) at 290 nm and flow-through radioactivity detection using solid scintillation detection (Ramona, Raytest, RSM Analytische Instrumente, Germany). The column (Zorbax XBD-C8; 5 µm; 150 by 4.6 mm, Agilent, USA) was developed using a 40 mL gradient from 100% buffer A (5% (v/v) methanol in water, 5 mM dimethylhexylamine, pH 8.1) to 20% buffer A and 80% buffer B (100% methanol, 5 mM dimethylhexylamine, pH 8.1) at a flow rate of 1 mL min^−1^ [53]. Radiolabeled methylene-tetrahydrofolate was used as a standard. It was synthesized from tetrahydrofolate (0.6 mg) and [^14^C]formaldehyde (14.8 kBq, 7 nmol) using the method of Heunnekens et al. [78].

### Incorporation of [carboxy-^14^C]4-aminobenzoate in *M. extorquens*

*M. extorquens* was grown aerobically in 1 L culture on mineral medium with methanol as substrate. In the exponential growth phase at OD_578 nm_ = 0.5, 370 kBq (173 nmol) [carboxy-^14^C]4-aminobenzoate was added. Samples (1 mL) were taken and radioactivity was determined in the culture as well as in cells and in the cell free supernatant; these fractions were obtained by centrifugation of the culture followed by two washing steps. The cells were harvested in the late exponential phase at OD_578_ = 2.5.

### Isolation of tetrahydropterins from ^14^C-labeled *M. extorquens*

All solvents contained 10 mM 2-mercaptoethanol. All purifications steps were carried out anaerobically mostly in an anaerobic glove box and under dim light. An aliquot of labeled frozen cells was mixed with 30 g unlabeled cells and suspended in 2-fold volume of 10 mM 2-mercaptoethanol. The suspension was boiled for 30 min under N_2_ gassing, centrifuged (30 min, 16,000×g at 4°C) and the pH of the supernatant was adjusted to 3.5 by addition of 600 mM ammonium formate buffer pH 3.5 to a final concentration of 60 mM. Approximately 15 mL of the supernatant was applied to a solid phase extraction (SPE) cartridge (STRATA; C_18_ (EC) 500 mg, 3 mL Phenomenex), which was preconditioned with 3 mL of 100% methanol and equilibrated with 3 mL of 60 mM ammonium formate buffer pH 3.5 (buffer 1). The cartridge was washed with 5% methanol in buffer 1. The pterin fraction was eluted with 90% methanol. Methanol in the pterin fraction was evaporated and the pH of the evaporated pterin fraction was adjusted to 5.5 using 0.1 M NaOH. Formaldehyde with a final concentration of 25 mM was added to the solution and the mixture was incubated at room temperature for 20 min. To prevent decomposition of methylene-tetrahydropterins the solution was kept under N_2_ atmosphere and the pH was adjusted to 9. The solution was analyzed by HPLC with UV and flow-through radioactivity detection. The column (LiChrospher 100 RP-C18e; 5 µm, 125 by 4 mm; Merck) was developed using a linear gradient (45 mL) from 100% buffer A to 20% buffer A and 80% buffer B at a flow rate of 1 mL·min^−1^.

### Mass spectrometry

ESI mass spectra were obtained on a Finnigan *LCQ Advantage* instrument.

Dir. neg. ESI (MeOH) = 859.3 (76%, [M-H]^+^); Dir. pos. ESI (MeOH) = 861.2 (7%, [M+H]^+^) Formyl-tetrahydromethanopterin tetraglutamate: Exact Mass M = 860.29.
